# Validation of surrogate markers for metabolic syndrome and cardiometabolic risk factor clustering in children and adolescents: A nationwide population-based study

**DOI:** 10.1371/journal.pone.0186050

**Published:** 2017-10-19

**Authors:** Ji-Young Seo, Jae Hyun Kim

**Affiliations:** 1 Department of Pediatrics, Nowon Eulji Medical Center, Eulji University, Seoul, Korea; 2 Department of Pediatrics, Seoul National University Bundang Hospital, Seongnam, Korea; Beijing Key Laboratory of Diabetes Prevention and Research, CHINA

## Abstract

Prevalence of metabolic syndrome (MetS) in children is increasing and identifying the risk factors for MetS during childhood is an important first step to prevent chronic diseases later in life. The aim of the present study was to evaluate the prevalence of MetS and cardiometabolic risk factor (CMRF) clustering among Korean children and adolescents and to validate the associated anthropometric and laboratory surrogate markers. We used data from the 2011–2014 Korean National Health and Nutrition Examination Survey. In total, data for 2,935 subjects (1539 boys, 52.6%) aged 10–19 years were assessed. MetS was defined by central obesity plus any two or more of CMRFs such as abdominal obesity, hypertension, hyperglycemia, hypertriglyceridemia, and decreased high density lipoprotein cholesterol (HDL-C) using the International Diabetes Federation criteria for children and adolescents. The presence of two or more CMRFs was classified as CMRF clustering. The prevalence of MetS and CMRF clustering in this group was found to be 1.8% and 8.9%, respectively. The receiver operating characteristic analysis of MetS and CMRF clustering, and the area under the curve (95% confidence interval) of surrogate markers revealed that the waist circumference to height ratio [0.960 (95% CI 0.959–0.960), cut-off 0.491] showed the highest predictability for MetS whereas triglyceride to HDL-C ratio [0.891 (95% CI 0.891–0.892), cut-off 2.63] showed the highest predictability for CMRF clustering. Long-term follow-up is needed for further validation.

## Introduction

The metabolic syndrome (MetS) is generally defined as a cluster of metabolically related cardiovascular risk factors. MetS is becoming a major public health issue globally, because individuals with MetS have higher risk of developing type 2 diabetes and cardiovascular diseases (CVD) than those without it [1]. Prevalence of MetS in children is less than that of in adults; however, as prevalence of childhood obesity increases so does MetS [2, 3]. Identifying the markers that can predict the emergence of MetS during childhood would be an important first step to prevent chronic diseases later in life. Cardiometabolic risk factor (CMRF) clustering indicated aggregation of several cardiometabolic risk factors, such as components of MetS. CMRF clustering have a tendency to tracking from childhood to adulthood, hence timely intervention in high-risk children may provide an early opportunity to decrease the progression to overt cardiovascular disease [4]. Recently, shifting the focus to CMRF is emphasized over the need to define a pediatric MetS [5]. However, data are not available regarding whether and how to best assess the individual risk for the presence of MetS and CMRF clustering in clinical pediatric practice [6, 7]. Several studies on the MetS and CVD have been conducted based on Korea National Health and Nutrition Examination Survey (KNHANES), which were mainly conducted for adults; however, recently, studies on the prevalence and degree of risk of MetS have also been performed in children [8–10]. Furthermore, most of the studies compared the prevalence of MetS based on the well-known predictors. In addition, detailed analysis on the most predictive factor of MetS and CMRF and the cut-off values are lacking.

The aim of this study was to evaluate the prevalence of MetS and CMRF clustering among Korean children aged 10–19 years, in addition to evaluating the validity of well-known and emerging anthropometric and laboratory markers, such as body mass index (BMI), waist circumference (WC) to height ratio (WHtR), triglyceride to high density lipoprotein cholesterol (TG/HDL-C) ratio, glycated hemoglobin (HbA1c) and elevated alanine transaminase (ALT).

## Materials and methods

### Study population and database

We used data from the Korea National Health and Nutrition Examination Surveys (KNHANES) (2011–2014). KNHANES represent a series of population-based, cross-sectional surveys that select a representative group by using a stratified, multi-stage sampling design according to geographic area, age, and gender. Detailed descriptions of the study design and data collection have been published [11]. In brief, 192 primary sampling units per year were extracted from the whole country during 2011–2014. Twenty households in each primary sampling unit were selected using systematic sampling. In the selected household, those aged 1 year or more were potential candidates for the survey, which consisted of a health interview, health examination and nutrition survey. The sampling weights were assigned for each participant and household to represent the whole Korean population. The response rate of the KNHANES was 80.8% in 2011–2012 and 78.3% in 2013–2014. Of the 32,144 participants, 3,813 participants aged 10–19 were selected. For one or more of the following reasons, a total of 861 subjects were excluded: no record of fasting time or fasting less than 8 hours (n = 415); no anthropometric data (n = 304); incomplete laboratory data (n = 845); no blood pressure measurement (n = 309). Thus, we had a final sample of 2,952 subjects (1,545 boys and 1,407 girls) for our analyses. The KNHANES was approved by Institutional Review Board of the Korea Centers for Disease Control & Prevention (KCDC) and the KCDC Bioethics Committee (approval number: 2011-02CON-06-C, 2012-01EXP-01-2C, 2013-07CON-03-4C, and 2013-12EXP-03-5C). Informed consent was obtained from all participants including children and adolescents and their legal guardian(s) or parent(s) before data collection for KNHANES. The present study protocol was approved from examination by the Clinical Examination Committee of Seoul Eulji Hospital of Eulji University (Institutional Review Board no. EMCIRB 17–27) and supported by EMBRI Grants 2012EMBRISN0002 from the Eulji University.

### Anthropometric and laboratory measurements

Height was measured using a stadiometer (Seca 225, Seca, Hamburg, Germany) to the nearest 0.1 cm. Weight was measured using an electronic balance (GL-6000-20, G-tech, Seoul, Korea) to the nearest 0.1 kg. BMI was calculated by dividing weight (kg) by height (m) squared. Height, weight, and BMI were converted to z-score for age and sex using the Korean reference [12]. Overweight and obesity was defined as having a BMI of 85-94^th^ percentile and ≥ 95^th^ percentile for corresponding age and sex, respectively. WC was measured at the midpoint between the lower borders of the rib cage and the iliac crest at the end of normal expiration. WHtR was calculated WC (cm)/height (cm). Plasma glucose, total cholesterol, HDL-C, TG, aspartate aminotransferase (AST) and ALT were measured using a Hitachi Automatic Analyzer 7600 (Hitachi, Tokyo, Japan). Non-HDL-C was calculated as follows: total cholesterol value—HDL-C value. TG/HDL-C ratio was calculated by TG over HDL-C. HbA1c was measured using high performance liquid chromatography (HLC-723G7; Tosoh, Tokyo, Japan), which is the certified method by the National Glycohemoglobin Standardization Program.

### Definition of metabolic syndrome and cardiometabolic risk factors

Metabolic syndrome was defined by the International Diabetes Federation (IDF) criteria for children and adolescents [13]. The presence of two or more CMRFs such as abdominal obesity, hypertension, hyperglycemia, hypertriglyceridemia, and decreased HDL-C was classified as CMRF clustering.

Abdominal obesity was defined by WC ≥ 90^th^ percentile using Korean waist reference data for those younger than 16 years of age. For boys and girls older than 16 years of age, a WC of more than 90 cm and more than 85 cm was used respectively to define central obesity based on Korean-specific WC cut-off points [14]. Hypertension was defined by systolic blood pressure (SBP) ≥ 130 mm Hg or diastolic BP (DBP) ≥ 85 mm Hg. Hyperglycemia was defined by fasting glucose ≥ 100 mg/dL, and hypertriglyceridemia was defined by fasting TG level ≥ 150 mg/dL. Decreased HDL-C was defined as HDL-C level of < 40 mg/dL for boys aged 10–19 years and girls younger than 16 years of age; for girls 16 years of age and above it was < 50 mg/dL. Elevated ALT was defined as ≥ 35 IU/L for boys and ≥ 24 IU/L for girls [15].

### Statistical analysis

Stata 14.2 (StataCorp LP, College Station, Texas, USA) was used for the statistical analysis. According to the design of KNHANES, appropriate weights for each sample were applied for the analysis. Data were stated as weighted mean ± standard error (SE) for the continuous variables or the number of cases with weighted percent. Total cholesterol, TG, HDL-C, non-HDL-C, and TG/HDL-C ratio were log-transformed and stated as geometric mean ± SE. Student t-test for the continuous variables and chi-square test for the categorical variables were used. Logistic regression analysis was performed to evaluate the association between the surrogate markers, MetS and CMRF clustering in addition to calculating the odds ratio (OR) with 95% confidence interval (CI). To validate surrogate markers as a predictor of multiple CMRFs and MetS, the area under the curve (AUC) was calculated from the receiver operating characteristic (ROC) curve [16]. The best cut-off point was determined using Youden index as [maximum (J = sensitivity + specificity—1)]; *P* < 0.05 was considered significant.

## Results

### Anthropometric, clinical, and biochemical characteristics of participants by gender

The general characteristics of the study subjects are shown in Table 1. The mean age of the subjects was 14.8 ± 0.1 years, and the mean BMI was 20.9 ± 0.1 kg/m^2^. Overall, 13.1% of the subjects (14.3% of boys and 11.9% of girls) were overweight and 12.8% of the subjects (15.0% of boys and 10.3% of girls) were obese. The prevalence of being overweight and obese was higher in boys. Furthermore, boys had elevated WC, WHtR, BP, fasting glucose, HbA1c, AST and ALT, whereas girls had elevated TC, HDL-C, and non-HDL-C levels.

**Table 1:** Anthropometric, clinical, and biochemical characteristics in study participants.

	Total (n = 2935, 100%)	Boys (n = 1539, 52.6%)	Girls (n = 1396, 47.4%)	*P* value
Estimated population	5,191,866	2,728,574	2,463,292	-
Age (years)	14.8 ± 0.1	14.8 ± 0.1	14.9 ± 0.1	0.265
Height (cm)	162.4 ± 0.2	166.3 ± 0.4	158.1 ± 0.2	<0.001
Height z-score	0.35 ± 0.03	0.41 ± 0.03	0.28 ± 0.04	0.003
Weight (kg)	55.9 ± 0.3	59.6 ± 0.5	51.9 ± 0.4	<0.001
Weight z-score	0.18 ± 0.03	0.20 ± 0.04	0.16 ± 0.04	0.343
Body mass index (kg/m^2^)	20.9 ± 0.1	21.2 ± 0.1	20.6 ± 0.1	<0.001
BMI z-score	0.02 ± 0.03	0.01 ± 0.04	0.04 ± 0.04	0.571
BMI classification				
Normal (%)	2210 (74.1%)	1114 (70.7%)	1096 (77.8%)	0.001
Overweight (%)	380 (13.1%)	211 (14.3%)	169 (11.9%)	
Obese (%)	345 (12.8%)	214 (15.0%)	131 (10.3%)	
Waist circumference (cm)	70.2 ± 0.2	72.2 ± 0.3	67.9 ± 0.3	<0.001
Waist circumference to height ratio	0.432 ± 0.001	0.434 ± 0.002	0.429 ± 0.002	0.041
Systolic blood pressure (mm Hg)	107.6 ± 0.2	110.0 ± 0.3	104.9 ± 0.3	<0.001
Diastolic blood pressure (mm Hg)	66.5 ± 0.2	66.9 ± 0.3	66.0 ± 0.3	0.019
Total cholesterol (mg/dL)	156.3 ± 0.7	151.5 ± 0.9	161.8 ± 0.9	<0.001
Triglyceride (mg/dL)	73.8 ± 0.9	73.1 ± 1.2	74.6 ± 1.3	0.358
HDL-C (mg/dL)	49.4 ± 0.3	47.6 ± 0.3	51.4 ± 0.4	<0.001
Non-HDL-C (mg/dL)	105.1 ± 0.7	102.4 ± 0.9	108.3 ± 0.9	<0.001
Triglyceride/HDL-C ratio	1.48 ± 0.03	1.51 ± 0.03	1.45 ± 0.03	0.186
Fasting glucose (mmol/L)	4.99 ± 0.01	5.01 ± 0.01	4.96 ± 0.02	0.042
HbA1c (%)	5.45 ± 0.01	5.46 ± 0.01	5.45 ± 0.01	0.441
Serum AST (IU/L)	18.6 ± 0.2	20.3 ± 0.2	16.8 ± 0.2	<0.001
Serum ALT (IU/L)	15.1 ± 0.3	18.1 ± 0.5	11.7 ± 0.2	<0.001
Metabolic syndrome (%)	31 (1.8%)	18 (1.9%)	13 (1.7%)	0.765
CMRF clustering (%)	188 (8.9%)	109 (9.4%)	79 (8.2%)	0.407
Abdominal obesity (%)	243 (9.1%)	119 (8.2%)	124 (10.2%)	0.109
Hypertension (%)	92 (3.6%)	70 (5.6%)	22 (1.5%)	<0.001
Hyperglycemia (%)	207 (7.0%)	128 (8.2%)	79 (5.6%)	0.015
Hypertriglyceridemia (%)	229 (8.6%)	133 (9.3%)	96 (7.9%)	0.254
Low HDL-C (%)	389 (18.2%)	207 (17.7%)	182 (18.7%)	0.614
Elevated ALT (%)	146 (5.1%)	102 (7.0%)	44 (3.1%)	<0.001

BMI: body mass index; HDL-C: high density lipoprotein cholesterol; aspartate transaminase; HbA1c: glycated hemoglobin; ALT: alanine transaminase; CMRF: cardio metabolic risk factor.

Data were expressed as weight mean ± standard error or number of cases (weighted percent).

Total cholesterol, triglyceride, HDL-C, non-HDL cholesterol and TG/HDL-C ratio were log-transformed and expressed as geometric mean ± standard error.

The prevalence of MetS and CMRF clustering were found as 1.8% and 8.9% respectively. There were no significant differences between boys and girls. Boys also had higher metabolic co-morbidities such as hypertension (5.6% in boys and 1.5% in girls), hyperglycemia (8.2% in boys and 5.6% in girls), elevated ALT (7.0% in boys and 3.1% in girls). However, there was no significant difference between boys and girls for abdominal obesity, hypertriglyceridemia, and low HDL-C (Table 1).

### Association between parameters and metabolic syndrome or CMRF clustering by gender

On multiple logistic regression analysis adjusted for age and gender, predictors for MetS were BMI z-score [OR 11.4 (95% CI 7.3–17.8), *P* < 0.01], HbA1c [OR 1.8 (95% CI 1.3–2.6), *P* < 0.01]. TG/HDL-C ratio [OR 2.3 (95% CI 1.8–3.0), *P* < 0.01] and WHtR [OR 1.4 (95% CI 1.3–1.5), *P* < 0.01] were also significantly associated with MetS. When we analyzed this factors by age group (10–15 years vs 16–19 years), similar analytic tendency was showed. There was no statistically significant gender difference in the prevalence of MetS with the increase of HbA1c, and the OR of BMI z-score was higher in boys as compared with girls (15.4 vs 8.9) (Table 2).

**Table 2:** Age- and sex-adjusted odds ratios (95% confidence intervals) of surrogate markers predicting metabolic syndrome and cardiometabolic risk factor (CMRF) clustering.

Category	Surrogate markers	Total	10–15 years	16–19 years	Boys	Girls
Metabolic syndrome	HbA1c (%)	1.8 (1.3–2.6)**	1.8 (1.3–2.7)**	1.7 (0.9–3.3)	3.88 (0.91–16.4)**	1.59 (1.08–2.35)**
	TG/HDL-C ratio	2.3 (1.8–3.0)**	1.9 (1.3–2.8)**	3.1 (2.1–4.5)**	2.11 (1.31–3.39)**	2.56 (2.00–3.28)**
	Non-HDL-C (mg/dL)	1.04 (1.03–1.05)**	1.03 (1.02–1.05)**	1.04 (1.03–1.06)**	1.03 (1.02–1.05)**	1.05 (1.03–1.06)**
	ALT (IU/L)	1.03 (1.02–1.05)**	1.03 (1.02–1.04)**	1.05 (1.03–1.07)**	1.03 (1.02–1.04)**	1.06 (1.03–1.10)**
	WHtR (%)	1.4 (1.3–1.5)**	1.4 (1.3–1.5)**	1.4 (1.3–1.5)**	1.37 (1.28–1.46)**	1.48 (1.35–1.62)**
	BMI z-score	11.4 (7.3–17.8)**	13.5 (6.7–27.2)**	10.5 (5.6–19.7)**	15.4 (7.1–33.3)**	8.9 (5.1–15.6)**
CMRF clustering	HbA1c (%)	2.1 (1.1–4.0)**	2.0 (0.9–4.7)	2.0 (0.8–5.2)	3.7 (1.7, 7.9)**	1.7 (1.0–3.0)*
	TG/HDL-C ratio	3.9 (3.3–4.6)**	4.2 (3.4–5.3)**	4.1 (3.1–5.3)**	3.7 (3.0–4.5)**	4.6 (3.4–6.1)**
	Non-HDL-C (mg/dL)	1.03 (1.03–1.04)**	1.04 (1.03–1.04)**	1.03 (1.02–1.04)**	1.03 (1.02–1.04)**	1.03 (1.02–1.04)**
	ALT (IU/L)	1.05 (1.03–1.06)**	1.05 (1.02–1.07)**	1.05 (1.04–1.06)**	1.04 (1.03–1.05)**	1.06 (1.02–1.11)**
	WHtR (%)	1.3 (1.3–1.4)**	1.3 (1.2–1.4)**	1.3 (1.2–1.4)**	1.3 (1.2–1.4)**	1.3 (1.3–1.4)**
	BMI z-score	4.1 (3.2–5.2)**	4.7 (3.4–6.5)**	3.6 (2.6–5.1)**	4.6 (3.1–6.9)**	3.6 (2.6–4.9)**

*P <0.05,

**P <0.01

HbA1c: glycated hemoglobin; TG/HDL-C: triglyceride to high density lipoprotein cholesterol; ALT: alanine transaminase; WHtR: Waist circumference to height ratio; BMI: Body mass index.

When CMRF clustering and surrogate markers were analyzed by the same method, HbA1c [OR 2.1 (95% CI 1.1–4.0), *P* < 0.01], BMI z-score [OR 4.1 (95% CI 3.2–5.2), *P* < 0.01], TG/HDL-C ratio [OR 3.9 (95% CI 3.3–4.6), *P* < 0.01] and WHtR [OR 1.3 (95% CI 1.3–1.4), *P* < 0.01] showed a high correlation with CMRF clustering overall, similarly to those of MetS (Table 2).

### Predictors for MetS and CMRF clustering

After ROC analysis of MetS and CMRF clustering, AUC of surrogate markers revealed that BMI z-score [0.959 (95% CI 0.957–0.962)] and WHtR [0.960 (95% CI 0.959–0.960)] showed the highest predictability for MetS, whereas TG/HDL-C ratio [0.891 (95% CI 0.891–0.892)] showed the highest predictability for CMRF clustering (Table 3). The cut-off values of WHtR were 0.491 (sensitivity 96.5% and specificity 88.5%) for MetS and 0.469 (sensitivity 74.1% and specificity 82.9%) for CMRF clustering (Table 3). BMI z-score showed the cut-off values of 1.35 (sensitivity 96.5% and specificity 88.5%) for MetS and 1.06 (sensitivity 68.2% and specificity 82.9%) for CMRF clustering. The cut-off points of TG/HDL-C ratio for predicting MetS and CMRF clustering were 2.64 (sensitivity 95.1% and specificity 86.4%) and 2.63 (sensitivity 74.4% and specificity 90.5%), respectively.

**Table 3:** Area under the curve (95% confidence intervals) and cut-off values of surrogate markers for predicting metabolic syndrome and cardiometabolic risk factor (CMRF) clustering.

Category	Surrogate markers	Total	10–15 years	16–19 years	Boys	Girls	Cut-off value	Sensitivity (%)	Specificity (%)
Metabolic syndrome (IDF)	HbA1c (%)	0.627 (0.625, 0.629)	0.637 (0.635, 0.639)	0.624 (0.621, 0.627)	0.621 (0.618, 0.623)	0.633 (0.630, 0.636)	5.5	70.5	50.5
	TG/HDL-C ratio	0.947 (0.946, 0.948)	0.965 (0.964, 0.965)	0.936 (0.934, 0.937)	0.934 (0.932, 0.935)	0.963 (0.962, 0.964)	2.64	95.1	86.4
	Non-HDL-C (mg/dL)	0.779 (0.778, 0.781)	0.747 (0.745, 0.749)	0.808 (0.806, 0.810)	0.735 (0.733, 0.737)	0.844 (0.842, 0.845)	111.6	81.3	63.7
	ALT (IU/L)	0.820 (0.819, 0.822)	0.816 (0.814, 0.818)	0.822 (0.820 0.823)	0.867 (0.865, 0.868)	0.811 (0.809, 0.813)	21	63.5	88.2
	WHtR	0.960 (0.959, 0.960)	0.954 (0.953, 0.954)	0.964 (0.963, 0.964)	0.967 (0.967, 0.968)	0.957 (0.957, 0.957)	0.491	96.5	88.2
	BMI z-score	0.959 (0.957, 0.962)	0.957 (0.956, 0.957)	0.962 (0.961, 0.962)	0.966 (0.966, 0.966)	0.955 (0.954, 0.955)	1.35	95.1	89.4
CMRF clustering	HbA1c (%)	0.607 (0.606, 0.608)	0.596 (0.594, 0.597)	0.628 (0.627, 0.629)	0.580 (0.578, 0.581)	0.636 (0.634, 0.637)	5.6	50.0	66.8
	TG/HDL-C ratio	0.891 (0.891, 0.892)	0.917 (0.916, 0.918)	0.869 (0.868, 0.869)	0.898 (0.898, 0.899)	0.885 (0.884, 0.885)	2.63	74.4	90.5
	Non-HDL-C (mg/dL)	0.720 (0.719, 0.721)	0.716 (0.715, 0.717)	0.725 (0.724, 0.726)	0.708 (0.707, 0.709)	0.738 (0.737, 0.739)	118.7	60.1	75.2
	ALT (IU/L)	0.730 (0.729, 0.730)	0.780 (0.779, 0.781)	0.686 (0.685, 0.687)	0.798 (0.797, 0.799)	0.704 (0.702, 0.705)	15	61.8	74.4
	WHtR	0.842 (0.841, 0.843)	0.843 (0.842, 0.844)	0.840 (0.839, 0.841)	0.857 (0.856, 0.858)	0.829 (0.828, 0.830)	0.469	74.1	82.9
	BMI z-score	0.829 (0.828, 0.830)	0.839 (0.838, 0.840)	0.821 (0.820, 0.822)	0.853 (0.852, 0.854)	0.804 (0.803, 0.805)	1.06	68.2	87.2

All *P* values were <0.001 when compared AUC between age groups and sex.

HbA1c: glycated hemoglobin; TG/HDL-C: triglyceride to high density lipoprotein cholesterol; ALT: alanine transaminase; WHtR: waist circumference to height ratio; BMI: body mass index.

## Discussion

In the present study using the 2011–2014 KNHANES, the prevalence of MetS and CMRF clustering in children and adolescents aged 10–19 years was 1.8% and 8.9%, respectively. The degree of risk was higher as the WHtR and TG/HDL-C ratio increased.

Overall, the prevalence of MetS among our participants (1.8%) was similar to previous studies based on the IDF criteria in 2007–2008 KNHANES (1.9%) and 2005 KNHANES (1.8%) [8, 9]. Compared to the prevalence of 1.5% shown in the 2007–2009 KNHANES study conducted in 2716 individuals aged 10–20 years [10], the prevalence of MetS seems to be increasing, but an accurate comparison cannot be made because the participants in these two studies belong to different age groups. On the other hand, prevalence of MetS in the present study was lower compared to the other studies of KNHANES conducted using the modified National Cholesterol Education Program (NCEP) in 1998, 2001, 2005, 2008, 2010–2014, where the prevalence was 7.5%, 9.8%, 10.9%, 6.7%, 6.2%, respectively [17, 18]. IDF guidelines state the criteria for hypertriglyceridemia as ≥ 150 mg/dL, which is higher than what the NCEP guidelines state (≥ 110 mg/dL). Furthermore, the criteria for low HDL-C is < 40 mg/dL for both genders, except in girls 16 and older (HDL-C < 50 mg/dL) in NCEP guidelines, which is stricter. Hence, using IDF guidelines criteria with higher cut-off point results in lower prevalence of hypertriglyceridemia [7].

When compared to the existing data from previous studies, where the IDF guidelines for MetS in children and adolescents [10, 19], we noted that the proportion of abdominal obesity increased in girls while hypertriglyceridemia decreased, and low HDL-C decreased in both genders (abdominal obesity: 7.7% in 1998–2008 KNHANES, 9.9% in 2007–2009 KNHANES, and 10.2% in the present study; hypertriglyceridemia: 9.7%, 9.2%, and 7.9%; low HDL-C 21.6%, 17.9%, and 17.7% in boys and 26%, 21.8%, and 18.5% in girls, respectively). In a study evaluating the changes in metabolic syndrome in American and Korean Youth from 1997 to 2008 [20], the WC in Korean youth has shown a tendency to increase. On the other hand, according to NCEP, MetS has been increasing because of the increased prevalence of low HDL-C, hypertriglyceridemia, and abdominal obesity, although it is evaluated based on different guidelines [20]. In any case, after 2008, the rates of low HDL-C and hypertriglyceridemia in Korean youth seem to be decreasing.

Meanwhile, it should be noted that the rate of hyperglycemia dramatically decreased in both genders when results from this study are compared to the study that used 1998–2008 KNHANES data (18.3% vs. 8.1% in boys; 15.2% vs 5.1% in girls) [19]. In addition, the rate of CMRF clustering decreased in both genders (10.9% vs. 9.3% in boys; 9.6% vs. 8.1% in girls) which seems to be the result of decreases in the rate of hypertriglyceridemia, low HDL-C and hyperglycemia. Although the rate of central obesity in the Republic of Korea and China is lower than that of the United States of America, the rates of low HDL-C and hypertriglyceridemia are similar [10, 21, 22]. The present study showed a similar pattern to previous study performed in U.S. children and adolescents (low HDL-C, 18.1% for Koreans vs. 22.6% for Americans; hypertriglyceridemia, 8.6% vs. 8.9%; central obesity, 9.1% vs. 28.6%).

The most well-known risk factor for MetS and CVD is obesity [21–23] and there are more studies being conducted in an effort to find more diverse surrogate markers, such as hypertriglyceridemia, hyperglycemia and high BMI [24, 25]. Analysis of MetS and surrogate markers in this study showed that as BMI z-score increased, the degree of risk became higher [OR 11.4 (95% CI 7.3–17.8, *P* < 0.001]. Cut-off values for BMI z-score predicting CMRF and MetS were 1.06 and 1.36, which corresponds 85^th^ percentile and 91^st^ percentile, respectively. This finding supports the recently published guidelines for pediatric obesity, that recommends performing screening test for comorbidities in children and adolescents with a BMI of ≥ 85^th^ percentile [26]. The value of OR was especially higher in boys as compared with girls (Table 2). Data for adults in KNHANES also shows a higher OR value in males [27]. Additionally, boys had higher attributable risk rates of metabolic co-morbidities as compared with girls [8]. In the analysis of CMRF clustering surrogate markers (Table 3), the degree of risk for HbA1c and BMI were high, and the OR value of HbA1c was higher in girls.

In the present study, the elevated ALT value was included in CMRF clustering. It was found that elevated ALT level was weak surrogate marker for MetS and CMRF clustering. However, excess adiposity can result in hepatic insulin resistance, hepatic steatosis, and Nonalcoholic fatty liver disease (NAFLD), well-known components of MetS [28, 29]. Some studies showed that low ALT level is associated with ideal cardiovascular health behavior [30] and MetS has been shown to have a dose-response relationship with ALT level [31]. However, these studies were mainly conducted on adults, so the basis for using ALT level as a surrogate marker for MetS in children seems insufficient.

AUC was the largest with the application of BMI z-score for MetS and WHtR for CMRF clustering among anthropometric factors. TG/HDL-C ratio showed the largest AUC for predicting MetS and CMRF clustering among laboratory surrogate markers (Table 3). Although there are some differences based on countries and races, cut-off point of 0.5 for WHtR is proposed as the universal cut-off for central obesity in both adults and children [32]. In a research paper that used 1998–2008 KNHANES data to analyze WHtR as an index of cardiometabolic risk, the optimal cut-off values for obesity screening was 0.51 in boys and 0.49 in girls, and in adolescents with central obesity, the rate of MetS was more than two times higher in those with WHtR ≥ 0.5, and concluded that the validity of WHtR to identify CMRFs was higher than that of BMI [19]. However, the study did not suggest any cut-off values for predicting MetS or CMRF clustering. In the present study, the cut-off value of WHtR was 0.491 (sensitivity 96.5% specificity 88.2%) for MetS and 0.469 (sensitivity 74.1% specificity 82.9%) for CMRF clustering. In studies using the 2010–2014 KNHANES data [18] on children where NCEP ATP III criteria were applied, the optimal cut-off WHtR value for predicting MetS risk was 0.44 in boys and 0.43 in girls. These values are lower than that of the present study and are also different than 0.52 value that was proposed by an American study [33]. In a study where the IDF guidelines were applied, the cut-off values were low (0.465 for boys, and 0.455 for girls); however, this seemed to be due to differences in race [34].

In the present study, the AUC of WHtR was higher, with a slight difference, than that of BMI in both MetS and CMRF clustering, with a value close to 1. As an anthropometric predictor of MetS, there were many studies showing that WHtR was similar or superior to BMI or WC [35–38]. However, some suggest that these be used as prescreening tools for predicting cardiometabolic risks, because anthropometric variables have low sensitivity [39]. We propose that in light of the inconvenience of using the percentile chart as a reference for BMI or WC of children and adolescents, WHtR might be an appropriate screening tool that can be used in clinical practice.

In contrast to children and adolescents, there were many studies that analyzed the validity or cut-offs of HbA1c or fasting glucose as predictive factors for diabetes, MetS, and cardiovascular diseases in adults [40–42]. In the present study, we found that HbA1c had less predictive value for predicting MetS and CMRF clustering than other markers. This may be because children and adolescents had a narrower range of HbA1c levels than adults.

In the ROC analysis of present study, the AUC of TG/HDL-C ratio was the highest among the laboratory markers. In a research on CVD risk analysis on adults, elevated TG/HDL-C ratio was a marker that reflected insulin resistance and glycemic control and that it was effective in MetS diagnosis to predict the development of CVD. In those studies, the value for high risk group of MetS was ranged to be ≥ 3.0–3.5 in males and ≥ 2.0 in females [43–46]. In a few studies done on the value of TG/HDL-C ratio in children, the mean TG/HDL-C ratio was 1.6–1.7 and 4.0 in the case of MetS, where the 95^th^ percentile values were 3.83–4.61 [47, 48]. The cut-off of 2.64 in MetS and 2.63 in CMRF clustering in the present study belonged to approximately 75-90^th^ percentile of Korean adolescents [48]. There are studies in which low cut-off values for MetS were TG/HDL-C ratio > 1.25 and this was proposed as a better index than homeostatic model assessment for insulin resistance (HOMA-IR) index [49]. However, there are not enough studies on children and adolescents on this suggesting that further studies are needed to develop a consensus.

There are several limitations to this study. First, this study is cross-sectional, making it difficult to explain causal relationships or describe clear mechanisms related to surrogate markers of MetS and CMRF clustering. Secondly, because the HOMA-IR, which is known to reflect insulin resistance best, was not obtained in this data, there was no comparison with HOMA-IR. Thirdly, this study only analyzed anthropometric and laboratory data, so the degree of risk for MetS depending on the difference in lifestyle could not be determined.

Despite these limitations, this study has several strengths. First, it is a nationwide epidemiologic study that found the prevalence of MetS and CMRF clustering and their predictors in children and adolescents. Secondly, to the best of our knowledge, this is the first study to suggest a cut-off value of WHtR and TG/HDL-C associated with the prevalence of MetS and CMRF clustering in the Korean children and adolescents.

## Conclusions

In conclusion, the prevalence of MetS and CMRF clustering was 1.8% and 8.9% in Korean children and adolescents. Most reliable predictors for MetS and CMRF clustering were WHtR in anthropometric parameters and TG/HDL-C ratio in laboratory markers. When TG/HDL-C ratio and waist-height ratio are compared, WHtR is significantly better in predicting MetS whereas TG/HDL-C ratio is significantly better in predicting CMRF clustering. Long-term follow-up is needed for further validation.
